# Phylogeography of *Nasutitermes corniger* (Isoptera: Termitidae) in the Neotropical Region

**DOI:** 10.1186/s12862-017-1079-8

**Published:** 2017-11-23

**Authors:** Amanda de Faria Santos, Tiago Fernandes Carrijo, Eliana Marques Cancello, Adriana Coletto Morales-Corrêa e Castro

**Affiliations:** 1 Instituto de Biociências, Letras e Ciências Exatas, Universidade Estadual Paulista “Júlio de Mesquita Filho” (UNESP), Cristóvão Colombo Street, 2265 - Jardim Nazareth, São José do Rio Preto, SP 15054-000 Brazil; 2 Laboratório de Biologia Evolutiva, Faculdade de Ciências Agrárias e Veterinárias, Universidade Estadual Paulista “Júlio de Mesquita Filho” (UNESP), Prof. Paulo Donato Castellane Access Way – Vila Industrial, Jaboticabal, SP 14884-900 Brazil; 3 Centro de Ciências Naturais e Humanas, Universidade Federal do ABC (UFABC), Arcurus Street, 3 - Jardim Antares, São Bernardo do Campo, SP 09606-070 Brazil; 4 Laboratório de Isoptera, Museu de Zoologia da Universidade de São Paulo (MZUSP), Nazaré Avenue, 481 - Ipiranga, São Paulo, SP 04263-000 Brazil

**Keywords:** Termite, Population genetics, Mitochondrial gene, 16S rRNA, Biogeography, Dispersal route

## Abstract

**Background:**

The Neotropical Region is known for its biodiversity and ranks third in number of known termite species. However, biogeographic and phylogeographic information of termites of this region is limited compared to other world geographic regions. *Nasutitermes corniger* is widely distributed in the region and is of considerable economic importance. The goal of this study was to describe the phylogeography of *N. corniger* in the Neotropical Region, to better understand its evolutionary processes.

**Results:**

The sampled populations of *N. corniger* showed high genetic variation. Results indicated strong geographic structure among *N. corniger* populations, with most haplotypes not broadly shared among separated locations. Phylogeographic analyses showed a dispersal route for *N. corniger* from Central America into South America via the Isthmus of Panama, with subsequent dispersal through the highlands east of the Andes and into eastern regions of the continent. The majority of haplotypes were limited in distribution to proximal regions, corresponding to particular biomes (Atlantic Forest, Amazonia, Chaco, Cerrado and Caatinga).

**Conclusions:**

*Nasutitermes corniger* is suggested to be a good model for biogeographic and phylogeographic studies in the Neotropical Region. This study clarified the phylogeographic history of *N. corniger* and can contribute to the understanding of biogeographic dispersion processes in the Neotropical Region.

**Electronic supplementary material:**

The online version of this article (10.1186/s12862-017-1079-8) contains supplementary material, which is available to authorized users.

## Background

The American tropics is known for its biodiversity, arising from its heterogeneity of habitats, hybrid biogeographic formation, and complex geological history, factors that influence the geographic distribution of species [1].

Research confirms faunal exchange between the Americas. South America, isolated from the beginning of the Tertiary, was reconnected to the northern continent through the elevation of the Isthmus of Panama during the Pliocene, allowing continental species dispersion. Research suggests dispersion of Neotropical biota from South America towards the north [2–6]. Two cenocrons – sets of taxa that share a biogeographic history – have been recognized: the “Old Southern” or “Ancient Neotropical” that dispersed from South to North America during the Cretaceous and Paleocene, and the more recent dispersal after the Isthmus of Panama was formed during the Pliocene and the Pleistocene, ~3.5 to 4.6 million years ago (Ma) [1].

The Neotropical Region ranks third in number of known termite species, with more than 650 described species [7]. Biogeographic and phylogeographic information of termites of this region is limited compared to other world geographic regions. For example, many studies have been conducted of termite phylogeography in the Neartic Region, mainly of *Reticulitermes* [8–11], as well as of European [12–14], Asian [15, 16], and Australian [17] species.

*Nasutitermes corniger* (Isoptera: Termitidae: Nasutitermitinae) is the most widely distributed species of *Nasutitermes* in the Neotropical Region. As a successful invasive termite, it has considerable economic impact [18]. Studies of its ecology [19], social behavior [20, 21], and taxonomy [22, 23] have been conducted.

The main goal of this study was to gain information on the phylogeography of termites in the Neotropical Region by investigating and describing genetic variation in *Nasutitermes corniger*. A further goal was to analyze how this variation was structured and distributed over the area of the species occurrence and how it reflects the evolution of *N. corniger*.

Mitochondrial DNA (mtDNA) was used due to its characteristics of maternal inheritance, rapid evolutionary rate, and high intraspecific polymorphism [24]. We used the 16S rRNA mitochondrial molecular marker, which, despite being more conserved than other mitochondrial genes, revealed representative genetic variations and consistently showed genetic differences among populations in previous tests.

## Methods

### Samples and laboratory procedures

The study used 230 soldiers (to facilitate accurate species identification) of *N. corniger,* collected throughout its distribution area (Additional file 1). Collections of samples were performed with permission issued by *Instituto Chico Mendes de Conservação da Biodiversidade* – ICMBio, agency responsible for environmental studies in Brazil (permission number 40673–8). Specimens were stored in 100% ethanol at the Isoptera collection of *Museu de Zoologia da Universidade de São Paulo*, Brazil. Total DNA was extracted from the head of one soldier per colony using the phenol-chloroform method [25]. For partial amplification of mitochondrial gene 16S rRNA, we used the oligonucleotides *LR-J-13007* [26] and *LR-N-1398* [27] in a PCR reaction in a final volume of 25 μL comprising 3 μL of each oligonucleotide at 5 pmol/μL, 4 μL of deionized water, 12.5 μL of PCR Master Mix (Promega), and 2.5 μL of DNA, mean concentration 15 ng/μL. This reaction was submitted to amplification under the following conditions: initial denaturation at 94 °C for 2 min followed by 35 cycles of denaturation for 1 min at 94 °C, annealing of 1 min at 50 °C, and extension of 75 s at 72 °C, followed by a final extension at 72 °C for 7 min [28]. The PCR product was treated with the NucleoSpin® Gel and PCR Clean-up kit (Macherey-Nagel) following manufacturer’s instructions. The DNA samples were sequenced by the BigDye reagent kit (Perkin-Elmer) in an automatic sequencer ABI 3730 XL DNA Analyzer (Applied Biosystems), according to manufacturer’s instructions. The sequencing reaction was submitted to the same amplification reaction conditions as the mitochondrial gene fragment.

### Data analysis

The nucleotide sequences were read using Chromas Lite v. 2.01 (Technelysium Ltd., 2005). We aligned the sequences using the ClustalW tool in BioEdit v.7.0.9.0 [29], followed by visual inspection, and conducted descriptive statistics analyses in DnaSP v.5.10.01 [30]. To quantify the variation in the mtDNA sequences, the following parameters were estimated: number of polymorphic or segregating sites (S), nucleotide diversity (π), average number of nucleotide differences (k), and haplotype diversity (Hd). Using MEGA v.6 [31], we estimated the mean percentage of each base in the composition of the mtDNA sequences.

We performed Fu’s Fs [32] and Tajima’s D [33] neutrality tests with DnaSP v.5.10.01 [30]. Significant negative values for Fu’s Fs are indicative of population expansion [32]. Significant negative values for Tajima’s D suggest purifying selection, lift effect, or population expansion. Positive values indicate stabilizing selection or population contraction [33].

We also used DnaSP v.5.10.01 to calculate mismatch distribution. This analysis allows discrimination among populations that remained stable over time (multimodal curves) and populations that underwent demographic expansion from a small founding population (unimodal curves) [34–37].

For the phylogeographic analysis, we performed the nested clade phylogeographic analysis (NCPA) from a haplotype network obtained using the software TCS v.1.21 [38], nested manually according to the method proposed by Templeton [39]. Using GeoDis v.2.5 [40], we calculated clade distance (Dc), nested clade distance (Dn), and interior-tip (I-T) for the clades formed in the haplotypic network. The parameters calculated and their significance were tested in the phylogeographic inferences key of Templeton [41]. For verification of results, we conducted the Mantel test using Alleles in Space v.1.0 [42]. Significant *r* values nearest to 1 indicate strong positive correlation between the two variables. Significant values nearest to −1 indicate strong negative correlation between the variables. Values of zero denote no linear correlation between the matrices.

We conducted the analyzes of molecular variance (AMOVA) using Arlequin v.3.11 [43], including all haplogroups observed in the haplotypic network.

For estimating divergence time, we conducted a Bayesian inference analysis of the obtained haplotypes. This analysis was generated through the program BEAST 1.8.0 [44] using the lognormal relaxed molecular clock. We used three calibration points through the dating of fossil records: a point at *Nasutitermes* including the haplotypes of *N. corniger* and a sequence of *N. guayanae*, dated to 18 Ma [45]; a point at Nasutitermitinae including the group *Nasutitermes* and a sequence of *Mironasutitermes shangchengensis*, dated to 30 Ma [46]; and a point at Termitidae including the group Nasutitermitinae and two sequences of *Spinitermes* species that was dated at 55 Ma [45, 47]. The best nucleotide substitution model found for this analysis was HKY + G + I [48], selected by MEGA v.6 [31] and based on the Bayesian Information Criterion [49].

## Results

### Descriptive statistics

After alignment and visual inspection, we obtained 230 (401 bp) partial sequences of the 16S rRNA gene of *N. corniger*. Among the sequences, we found 33 polymorphic sites and 45 haplotypes with high haplotype diversity (Hd = 0.880). Nucleotide diversity was 0.00505 and the mean number of nucleotide differences was 1.988. The mean nucleotide composition of the sequences was 25.2% thymine (T), 21.1% cytosine (C), 42.7% adenine (A), and 11% guanine (G). These data showed high genetic variation among populations. The percentage of A/T bases was higher than the percentage of C/G bases, as is expected for insect mitochondrial genomes.

### Haplotypic network and phylogeographic analyses

**Fig. 1:**
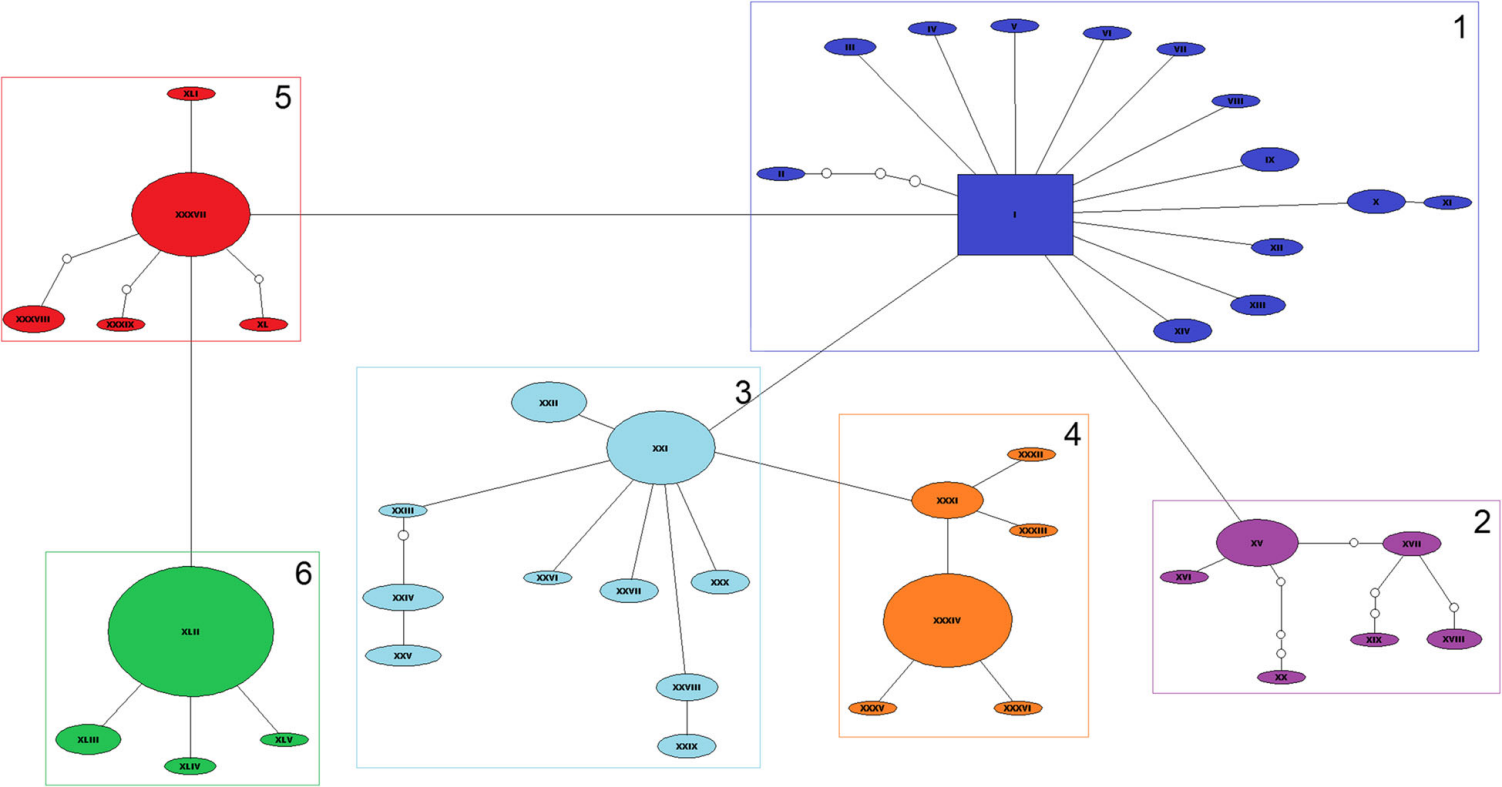
Haplotypic network of *N. corniger* generated through mitochondrial gene 16S rRNA sequencing. Legend: Each line in the network represents a single mutational step. Haplotypes are represented by Roman numerals within the ellipses, with the central haplotype represented by a rectangle. Small circles indicate hypothetical haplotypes that are necessary intermediates among the identified haplotypes, but which were not observed in the sampling. The rectangle in dark blue indicates haplogroup 1, purple = haplogroup 2, light blue = haplogroup 3, orange = haplogroup 4, red = haplogroup 5, and green = haplogroup 6

To observe the relationship among haplotypes, we generated a network using all analyzed sequences. The network (Fig. 1) grouped all specimens of *N. corniger* according to haplotype (Additional file 2), showing the proximity among them based on the number of mutational steps. We defined six large haplogroups in the network, distributed throughout the Neotropical Region. To establish the haplogroups, we observed the presence of frequent haplotypes positioned more centrally in their respective haplogroup, which gave rise to less frequent derived haplotypes positioned peripherally. This type of relationship among haplotypes produces a star-shaped structure, with several derived haplotypes around a central haplotype.

For the phylogeographic analyses among populations, we considered 19 clades in the NCPA (Additional file 3). Eleven clades showed significant results, including levels three and four (Table 1). Interpretation of the NCPA results using the Templeton Inference Key [41] frequently indicated “restricted gene flow with isolation-by-distance” as the source of the observed haplotype distribution pattern.

**Table 1:** Phylogeographic analysis of *N. corniger* based on NCPA and interpreted using the Templeton Inference Key [41]

Clade	Significant subset	Significant parameters	Significant I-T parameters	Phylogeographic inference
1–1	I (interior)	Dc>; Dn>	Dc>; Dn>	Restricted gene flow with isolation by distance
	IX (tip)	Dc<		
	XIV (tip)	Dc<		
1–9	XXII (tip)	Dc<	Dc>	Restricted gene flow with isolation by distance
	XXVII (tip)	Dc<		
	XXX (tip)	Dc<		
1–19	XLIII (tip)	Dc<	None	Restricted gene flow with isolation by distance
2–1	1–1 (interior)	Dc>; Dn>	Dc>; Dn>	Restricted gene flow with isolation by distance
	1–3 (tip)	Dc<; Dn<		
2–5	1–12 (tip)	Dc<	Dc>	Restricted gene flow with isolation by distance
2–7	1–13 (interior)	Dc>; Dn>	Dc>; Dn>	Restricted gene flow with isolation by distance
	1–14 (tip)	Dc<; Dn<		
2–8	1–19 (tip)	Dc<; Dn<	Dc>; Dn>	Restricted gene flow with isolation by distance
3–1	2–1 (interior)	Dc<	Dc>	Restricted gene flow with isolation by distance
	2–8 (tip)	Dc<		
3–2	2–4 (tip)	Dn<	Dn>	Restricted gene flow with isolation by distance
3–3	2–7 (tip)	Dc<	None	Restricted gene flow with isolation by distance
4–1	3–2 (tip)	Dc<; Dn>	Dc>; Dn>	Restricted gene flow or Dispersion with some long-distance dispersion
	3–3 (tip)	Dc<; Dn<		

*Dc* clade distance, *Dn* nested clade distance, *I-T* interior-tip

### Mantel test

The Mantel test was used to confirm the results of the NCPA. The test resulted in a significant positive R value (0.252090, *p* = 0.0009), indicating correlation between haplotype genetic distance and geographic distance, that is the number of differences observed among haplotypes from proximal localities was lower than among haplotypes from more widely separated sites.

### Neutrality tests and mismatch distribution

Neutrality tests and mismatch distribution analysis were performed to investigate the demographic history of the sampled populations. Initially, all *N. corniger* specimens were treated as a single group in the neutrality tests. The obtained Fu’s Fs and Tajima’s D values were significantly negative (−31.673; *p* < 0.05 and −3.3115; p < 0.05, respectively), indicating population expansion. Neutrality tests were also conducted separately for each of the six haplogroups (Table 2). All tests produced significant negative values of Fu’s Fs, again consistent with population expansion. Tajima’s D test produced a significant negative value only for haplogroup 1 (−1.96222; *p* < 0.05).

**Table 2:** Neutrality tests (DnaSP v.5). *N. corniger* specimens analyzed as a single group and with each haplogroup in the haplotype network analyzed separately (Fig. 1)

Samples considered	Fu’s *Fs*	Tajima’s D
All	−31.673*	−3.3115*
Haplogroup 1	−9.162*	−1.96222*
Haplogroup 2	−1.282*	−1.71862
Haplogroup 3	−2.787*	−0.37922
Haplogroup 4	−0.986*	−0.63467
Haplogroup 5	−0.883*	−1.43621
Haplogroup 6	−1.691*	−1.02028

*Significant values (*p* < 0.05)

**Fig. 2:**
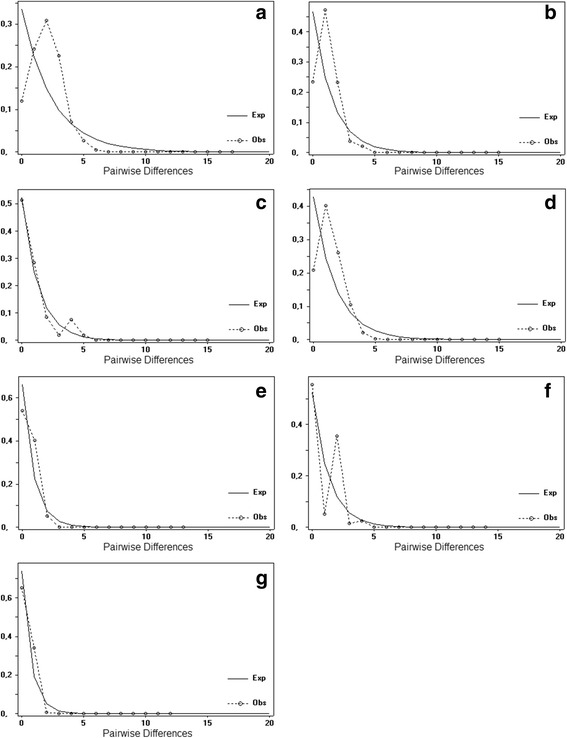
Pairwise difference mismatch distribution of the 16S rRNA sequences. Legend: Graph generated **a** for all *N. corniger* sampled populations; **b**-**g** for Haplogroups 1, 2, 3, 4, 5, and 6, respectively

We also analyzed mismatch distribution of all sequences of *N. corniger* as a single group well as of each haplogroup separately. The obtained graph showed unimodal curves, indicating that the populations may have undergone recent expansion (Fig. 2).

### Analysis of molecular variance (AMOVA)

An AMOVA was performed to evaluate the degree of genetic variation among *N. corniger* haplogroups. We tested two hierarchy constructions, the first including all haplogroups within a single larger group. The results of this analysis were significant (*p* < 0.05) and showed high genetic differentiation within and among the haplogroups (Table 3). The F_ST_ value indicated a high degree of genetic structure among the haplogroups.

**Table 3:** AMOVA assessment of genetic variation among*N. corniger* considering all haplogroups as a single group

Source of variation	Variance components	Variation %	Fixation index
Among haplogroups	0.99576	65.46	F_ST_ = 0.65457*
Within haplogroups	0.52547	34.54	–

*Significant value (*p* = 0.00000)

**Fig. 3:**
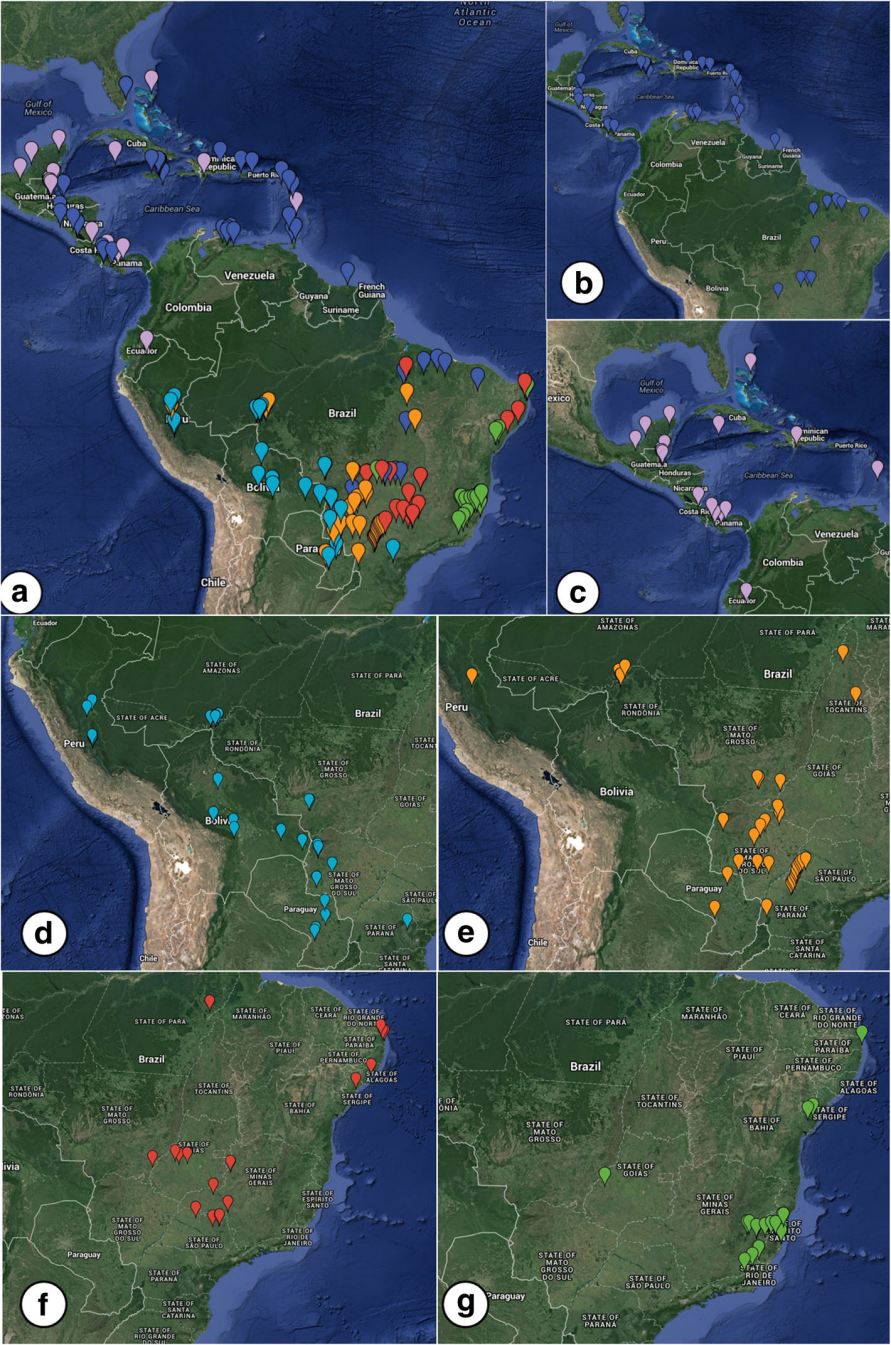
*Nasutitermes corniger* haplotype distribution. Legend: **a** Haplotypes allocated to their respective locations. The colors of the point markers correspond to the colors of the haplogroups observed in the network (Fig. 1); **b**-**g**: locations corresponding to the haplogroups 1, 2, 3, 4, 5, and 6, respectively. Images generated using Google Maps

In the second construction, we segregated the haplogroups according to geographic location to evaluate the degree of structure of haplogroups with restricted distribution. Haplogroup 1 was omitted from the analysis due to its broad distribution (Fig. 3). We constructed three larger groups, the first containing all sequences present in Haplogroup 2 (almost exclusively Central America), the second comprising sequences present in Haplogroups 3 and 4 (predominantly west-central South America), and the third containing the sequences of Haplogroups 5 and 6 (predominantly east-central South America). All results were significant (*p* < 0.05) and indicated population structure as shown by high F indices for the established groups (Table 4). The F_ST_ value (0.71436) indicated high genetic differentiation within haplogroups too.

**Table 4:** AMOVA assessment of genetic variation among the sampled *N. corniger* populations

Source of variation	Variance components	Variation %	Fixation index
Among groups	0.57593	32.72	F_CT_ = 0.32721*
Among haplogroups within groups	0.68143	38.71	F_SC_ = 0.57544*
Within haplogroups	0.50277	28.56	F_ST_ = 0.71436*

*Significant value (*p* = 0.00000)

### Analysis of divergence time

**Fig. 4:**
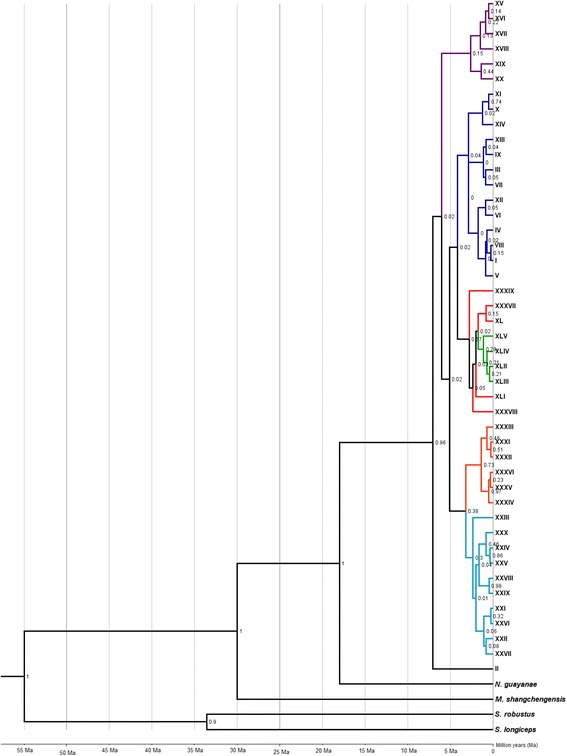
Bayesian tree generated with the 45 *Nasutitermes corniger* haplotypes obtained. Legend: The colors of the branches correspond to the colors of the haplogroups identified in the network (Fig. 1). Numbers at the nodes of branches correspond to Bayesian posterior probabilities. The bar below the tree marks the estimated divergence time

The divergence time of the haplogroups was estimated by Bayesian analysis (Fig. 4) using the 45 haplotypes obtained in this work. The topology of the tree confirmed the haplogroups observed in the network (Fig. 1) with the exception of Haplogroup 5, the branch of which includes Haplogroup 6. The tree provided evidence of two cladogenetic events generating the *N. corniger* haplogroups identified in this study. The first event corresponded to the divergence of the lineage that gave rise to Haplogroup 2, about 5.99 Ma. This was the first lineage to diverge, separate from the lineage of the five other haplogroups. The second cladogenetic event corresponded to the divergence among the lineages that gave rise to Haplogroups 1, 5, and 6, and to Haplogroups 3 and 4, about 5.1 Ma. However, although Haplogroup 2 was derived from the original lineage, its haplotypes diversified later than did those of Haplogroup 1. The diversification time for the haplotypes of Haplogroups 1 and 2 was 2.87 Ma and 2.62 Ma, respectively. That is, although the lineage of Haplogroup 1 was established later than Haplogroup 2, its haplotypes diverged earlier than those of Haplogroup 2. The diversification time of the branch that included Haplogroups 5 and 6 is the second oldest, at 2.77 Ma. The most recent estimated divergence time was 1.09 Ma for Haplogroup 6. The estimated diversification times for Haplogroups 3 and 4 were 2.33 Ma and 1.36 Ma, respectively.

## Discussion

### Genetic population results

The results indicated high genetic variation among the *N. corniger* sampled, as well as strong geographic structure among the populations.

The variation can be confirmed by a high number of haplotypes and high haplotype diversity. The structure was demonstrated by the configuration of the haplogroups in the network, which included primarily adjacent or closely related geographic regions, except for Haplogroup 1, which was more widely distributed. This pattern of genetic structure can also be shown by the high genetic distance among the haplogroups and, chiefly, by the AMOVA results of high F values. Most of the haplotypes identified in the *N. corniger* populations were not broadly shared among specimens from widely separated geographic areas.

The observed structure pattern demonstrated that haplotypes can be associated with geographic region. In Central America and the Caribbean Islands, we found only haplotypes of Haplogroups 1 and 2 (Figs 3a–c). Haplogroup 2 is essentially exclusive to these regions. The eastern regions of the Andes were characterized by Haplogroup 3 (Fig. 3d); in southwest Brazil, Haplogroup 4 predominated (Fig. 3e); in south-central Brazil, Haplogroup 5 (Fig. 3f); and in east-southeast Brazil we observed only Haplogroup 6 (Fig. 3g).

### Phylogeographic and biogeographic results

Phylogeographic analysis suggested a dispersal route of *N. corniger* from Central America toward South America via the Isthmus of Panama with subsequent dispersal through the highlands east of the Andes and eastern regions of the continent.

In the haplotypic network (Fig. 1), we observed that Haplotype I, included in Haplogroup 1, which occupied the central position, was common and widely distributed throughout the sampling area. The coalescence theory [50, 51] suggests that Haplotype I may be the most ancient haplotype. Thus, it was possible to identify a probable dispersion center for the species in Central America in which Haplotype I and others of this haplogroup are most common.

The Bayesian tree (Fig. 4) showed the diversification time of Haplogroups 1 and 2 to be close, 2.87 Ma and 2.60 Ma, respectively. Haplotypes of the two groups are found in both the continental areas and the islands of Central America. This suggests that migration of *N. corniger* in Central America and the Caribbean Islands occurred before the vicariance processes separating these regions. The times of these migration events are likely earlier than the diversification times of Haplogroups 1 and 2 estimated by Bayesian analysis.

The relationships of origin and descent among *N. corniger* haplotypes can be best understood via the haplotypic network, which is an appropriate method for making phylogeographic inferences. Bayesian analysis is efficient in phylogenetic analyses and accurate for the estimate of divergence and diversification times; however, it is of limited effectiveness in clarifying ancestral relations among intraspecific haplotypes [52, 53].

The haplotypic network (Fig. 1) in combination with the distribution patterns of haplotypes in the sampled regions (Fig. 3), allowed speculation of an *N. corniger* dispersion route. We assumed that the elevation of the Isthmus of Panama during Pliocene allowed dispersion of *N. corniger* toward South America. This event also allowed the exchange of many other faunistic taxa [3–6]. The diversification dates of Haplogroups 3, 4, 5, and 6, found only in South America, is later than the estimated date of complete elevation of the Isthmus of Panama, from 3.5 to 4.6 Ma, supporting the hypothesis that the event may have allowed dispersal of *N. corniger* populations to South America. A model of dispersal from North America to South America by terrestrial connection had been proposed for flies of the genera *Coenosopsia* (Diptera: Anthomyiidae) and *Phaonantho* (Diptera: Anthomyiidae) [54]. We suggest a similar pattern of dispersion for *N. corniger*, although at the specific level.

**Fig. 5:**
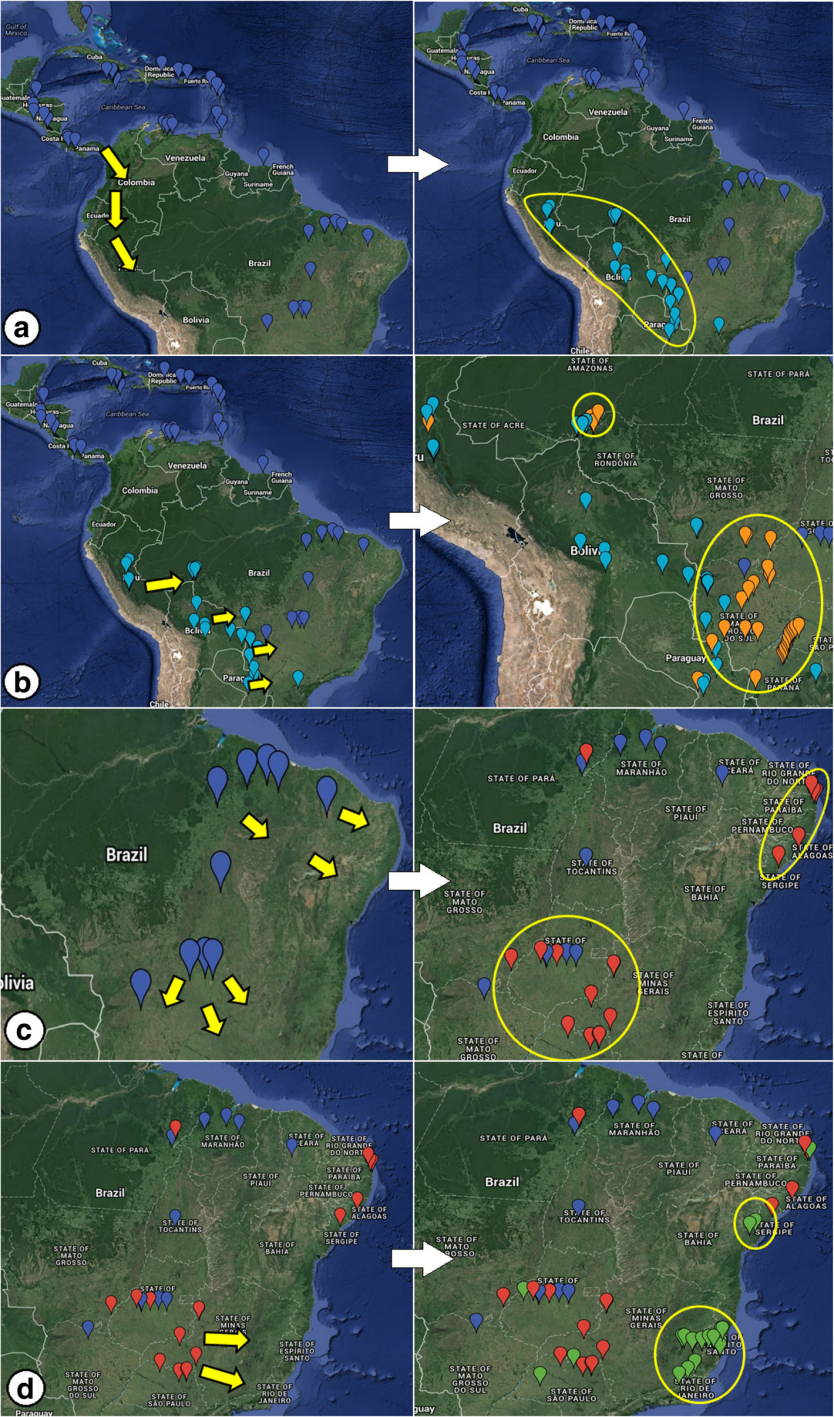
Dispersion processes of *Nasutitermes corniger* in South America. Legend: Establishment of Haplogroups 3, 4, 5, and 6 (**a** to **d**, respectively). Arrows indicate the direction of dispersion; circles show the region in which the haplogroups predominate. Images generated using Google Maps

The configuration of the haplogroups in the network (Fig. 1), the distribution of the haplotypes in the sampled regions (Fig. 3), and the diversification times of haplogroups suggest the following phylogeographic movements of *N. corniger* populations in South America:

1. After entering the South American continent, the populations dispersed in a southerly direction east of the Andes, originating the haplotypes of Haplogroup 3 about 2.62 Ma ago (Fig. 5a).
2. Populations that contained haplotypes of Haplogroup 3 dispersed in an easterly direction, originating Haplogroup 4 approximately 1.36 Ma ago, primarily in western Brazil. Some of these haplotypes reached the southwest portion of the State of São Paulo, Brazil (Fig. 5b).
3. Some haplotypes of Haplogroup 1 are found in central Brazil. It is probable that these haplotypes gave rise to Haplogroup 5, which is predominantly found in central and northeast Brazil (Fig. 5c).
4. Some haplotypes of Haplogroup 5 dispersed toward eastern Brazil, giving rise to Haplogroup 6, about 1.09 Ma (Fig. 5d).

Other models of dispersal routes in South America through the highlands of the east of Andes have been proposed [55]. A well-supported hypothesis states that organisms disperse naturally prior to becoming geographically isolated due to geological events, resulting in vicariance, for example [56]. This can explain the patterns observed in *N. corniger*. Throughout dispersal of *N. corniger* populations, some haplotypes may have differentiated and remained relatively isolated, resulting in a pattern of population structure caused by isolation-by-distance, as supported by results of the AMOVA and NCPA.

The geographic distribution of the *N. corniger* haplogroups and the biomes found in South America show overlap of some characteristics. Haplogroup 6 is essentially exclusive to the Atlantic Forest regions of southeast and northeast Brazil, suggesting that the biogeographic processes of formation and evolution of the Atlantic biome led to its genetic structure. The expansion of the diagonal of open formations (Chaco, Cerrado, and Caatinga), for example, isolated the Atlantic Forest from other South American forests [57] and contributed to its complex biogeographic history. Many areas of endemism have been proposed in the Atlantic Forest. The definition of these areas depends of the taxa studied, the variables evaluated, and the scale of the sampling [58].

We can associate the geographic location of Haplogroup 5 to the Cerrado and Caatinga areas, typically arid biomes of Brazil. The region also includes Haplogroup 1, suggesting residual populations that maintained earlier haplotypes. The occurrence of Haplogroups 5 and 6 in northeastern Brazil can be explained by areas of transition between the Atlantic biome, where Haplogroup 6 is nearly exclusive, and the Caatinga, where we found Haplogroup 5.

We observed a predominance of Haplogroups 3 and 4 in the Chaco region. Haplogroup 3 inhabits the transition zone between the Andean region and the Neotropical sensu stricto [1] reaching the Chaco in Bolivia and Paraguay. Haplogroup 4 included an area of Amazonian biome in Rondonia State, Brazil, and also included the Chaco, reaching easterly into regions of mixed forest (Interior Atlantic Forest) in Parana, Brazil.

## Conclusion

The sampled populations of *N. corniger* showed high genetic variation and structure throughout the Neotropical Region. In the haplotypic network, we observed six large haplogroups, which served as the basis for analyses of the evolutionary history of populations throughout their distribution area. From these analyses, it was possible to propose a dispersal route for the species, from Central America toward South America via the Isthmus of Panama and, subsequently, into the highlands of the eastern Andes, and eastern regions of the continent. New haplotypes were generated, many restricted to limited geographic areas corresponding to certain biomes, such as the populations found in the Atlantic Forest and in the open vegetation areas of Cerrado and Caatinga.

We suggest *Nasutitermes corniger* to be a good model for biogeographic and phylogeographic studies in the Neotropical Region. The use of temporal information provided by phylogenetic and phylogeographic molecular analyses, allow inference of associations between evolutionary pattern formation and a shared history of biotic diversification. This work helps to clarify the phylogeographic history of *N. corniger*, and can contribute to understanding of biogeographic dispersion processes in the Neotropical Region. We suggest that results of analysis by different models can be complementary in explaining patterns of speciation and diversity [59].

## Additional files

Additional file 1: Information of the *Nasutitermes corniger* specimens used. Voucher number, location code, locality of origin, and geographic coordinates of the *Nasutitermes corniger* specimens used in this work. (DOCX 37 kb)

Additional file 2: Details of observed *Nasutitermes corniger* haplotypes. Relationship among haplotypes, their frequency, sharing with *N. corniger* specimens, and allocation to haplogroup. Letters preceding voucher numbers (final column) correspond to the respective location code. (DOCX 19 kb)

Additional file 3: Haplotypic network evidencing the nested clades used in the NCPA. Each line of the network represents a single mutation step. Haplotypes are represented by Roman numerals; small circles indicate hypothetical haplotypes that are necessary intermediates between the haplotypes observed. Dotted lines indicate level 1 clades; dashed lines indicate level 2 clades; full clear lines indicate level 3 clades; full dark lines indicate level 4 clade. (PDF 218 kb)

Additional file 4: Dataset used in this study. Nucleotide sequences of the specimens used in this study. (TXT 110 kb)

## Abbreviations

A: Adenine
AMOVA: Analysis of molecular variance
BIC: Bayesian information criterion
C: Cytosine
DNA: Deoxyribonucleic acid
G: Guanine
Ma: Millions of years
MZUSP: Museu de Zoologia da Universidade de São Paulo
NCPA: Nested clade phylogeographic analysis
PCR: Polymerase chain reaction
T: Thymine
UFABC: Universidade Federal do ABC
UNESP: Universidade Estadual Paulista
