# Supplementary material for: Phylogeography of Nasutitermes corniger (Isoptera: Termitidae) in the Neotropical Region
Source: BMC Evol Biol. 2017 Nov 23;17:230. doi: 10.1186/s12862-017-1079-8 (PMC5701342; doi:10.1186/s12862-017-1079-8)
Supplement: Supplementary file 1 — Information of the Nasutitermes corniger specimens used. Voucher number, location code, locality of origin, and geographic coordinates of the Nasutitermes corniger specimens used in this work. (DOCX 37 kb) [file 12862_2017_1079_MOESM1_ESM.docx]

Information about the specimens used in this work.

| **Voucher number** | **Locality code** | **Locality of origin** | **Coordinates** | |
| --- | --- | --- | --- | --- |
|  |  |  | **Latitude** | **Longitude** |
| 88-95 | JAB | Jaboticabal - SP | -21.25472 | -48.32222 |
| 156 | RO | Porto Velho - RO | -9.14664 | -64.50953 |
| 158 | RO | Porto Velho - RO | -9.45261 | -64.39004 |
| 159 | RO | Porto Velho - RO | -9.45205 | -64.38508 |
| 161 | RO | Porto Velho - RO | -9.45022 | -64.36745 |
| 162 | RO | Porto Velho - RO | -9.44910 | -64.35772 |
| 163 | RO | Porto Velho - RO | -8.84126 | -64.06220 |
| 164 | RO | Porto Velho - RO | -8.83823 | -64.06554 |
| 165 | RO | Porto Velho - RO | -9.60888 | -65.37693 |
| 166 | RO | Porto Velho - RO | -9.61876 | -65.44740 |
| 167 | RO | Porto Velho - RO | -9.61162 | -65.45280 |
| 168 | RO | Porto Velho - RO | -9.43718 | -64.83201 |
| 170 | RO | Porto Velho - RO | -9.59153 | -65.05023 |
| 171 | RO | Porto Velho - RO | -9.57909 | -65.05786 |
| 172 | RO | Porto Velho - RO | -9.57274 | -65.06102 |
| 173 | RO | Porto Velho - RO | -9.59837 | -65.04759 |
| 174 | RO | Porto Velho - RO | -9.61621 | -65.04501 |
| 175 | RO | Porto Velho - RO | -9.59471 | -65.06456 |
| 176 | RO | Porto Velho - RO | -9.57405 | -65.07168 |
| 177 | RO | Porto Velho - RO | -9.60185 | -65.05691 |
| 178 | RO | Porto Velho - RO | -9.60185 | -65.05691 |
| 179-180 | MS | Corumbá - MS | -19.57680 | -57.01800 |
| 181-182 | MS | Corumbá - MS | -19.30605 | -57.05536 |
| 183 | MS | Corumbá - MS | -19.46908 | -57.02810 |
| 184-185 | MS | Corumbá - MS | -19.48230 | -56.99310 |
| 194 | RDOM | República Dominicana (AY623100.1*) | 19.221 | -69.529 |
| 195 | PRICO | Porto Rico (AY623098.1*) | 18.283 | -67.167 |
| 197 | MEX | México (AY623094.1*) | 17.449 | -92.104 |
| 198 | GUA | Guadalupe (AY623099.1*) | 16.167 | -61.664 |
| 199 | SKN | Saint Kiss e Nevis (AY623097.1*) | 17.13422 | -62.60183 |
| 200 | SUR | Suriname (AY623095.1*) | 5.86 | -55.15 |
| 201 | DOM | Dominica (AY623093.1*) | 15.58 | -61.32 |
| 202 | EQUA | Equador (AY623085.1*) | -1.90 | -77.83 |
| 203 | WIND | West Indies (AY486438.1*) | 17.0000 | -76.0000 |
| 267-269 | PR | Castro - PR | -24.79430 | -50.01594 |
| 270-274 | MT | Cáceres - MT | -16.02350 | -57.66110 |
| 275-279 | MT | Cáceres - MT | -16.00293 | -57.71705 |
| 280 | PB | João Pessoa - PB | -7.13445 | -34.84602 |
| 281 | PB | João Pessoa - PB | -7.13445 | -34.84602 |
| 282 | PB | João Pessoa - PB | -7.13445 | -34.84602 |
| 283 | MG | Uberlândia - MG | -18.9113 | -48.2622 |
| 297-298 | PB | Santa Rita - PB | -7.11631 | -34.9812 |
| 299 | CE | Tauá - CE | -6.1144 | -40.4477 |
| 300 | GO | Avelinópolis - GO | -16.5133 | -49.7372 |
| 301 | MA | Bom Jesus das Selvas - MA | -4.3133 | -46.5289 |
| 302-303 | MA | Lago Verde - MA | -4.022 | -45.0164 |
| 304 | MA | Codó - MA | -4.4575 | -44.1329 |
| 306 | PA | São João do Araguaia - PA | -5.4739 | -48.7946 |
| 307 | PA | Abel Figueiredo - PA | -4.8625 | -48.5467 |
| 309 | TO | Brejinho de Nazaré - TO | -11.0164 | -48.5689 |
| 310 | TO | Ponte Alta do Tocantins - TO | -10.7436 | -47.5378 |
| 311 | TO | Colinas do Tocantins - TO | -7.815 | -48.4529 |
| 347-348 | MG | Sacramento - MG | -20.2019444 | -47.122222 |
| 349 | AL | Maceió - AL | -9.8586111 | -35903888 |
| 350 | SE | Acaraju - SE | -10.929166 | -370475 |
| 351 | SP | Tanabi - SP | -20.619777 | -49.649185 |
| 352 | PGUAI | Caacupé – Paraguai | -25.38044 | -57.20014 |
| 353 | PGUAI | Guayaibi – Paraguai | -24.44771 | -56.43303 |
| 354 | PGUAI | Tacuati – Paraguai | -23.42052 | -56.49494 |
| 355 | PGUAI | Concepción – Paraguai | -23.05027 | -56.72764 |
| 356 | PGUAI | Asuncion – Paraguai | -25.27856 | -57.63545 |
| 357 | PGUAI | Yaguarón – Paraguai | -25.56204 | -57.28416 |
| 358 | PMA | Santiago – Panamá | 8.15942 | -81.05536 |
| 359 | PMA | Las Lajas – Panamá | 8.17263 | -81.86517 |
| 360 | PMA | Punta Robalo – Panamá | 9.04350 | -82.29374 |
| 361 | PMA | Canas Gordas – Panamá | 8.63203 | -82.82654 |
| 362 | PMA | Santa Marta – Panamá | 8.33266 | -82.67223 |
| 363 | PMA | Lajas de Tole – Panamá | 8.18740 | -81.72511 |
| 364 | PMA | Los Piedras – Panamá | 8.62902 | -80.48495 |
| 365 | PRU | Satipo – Peru | -11.28681 | -74.67691 |
| 366 | PRU | Rio Negro – Peru | -11.18070 | -74.68987 |
| 367 | PRU | Rio Negro – Peru | -11.18987 | -74.66985 |
| 368 | PRU | Bajo Pichanaqui - Peru | -11.06414 | -74.71955 |
| 369 | PRU | Pto. Inca - Peru | -9.46759 | -75.02367 |
| 370 | PRU | Pucallpa – Peru | -8.36714 | -74.71778 |
| 371-372 | PRU | Von Humbolt - Peru | -8.85449 | -75.11904 |
| 373 | BOL | Flora & Fauna Hotel - Bolívia | -17.49893 | -63.65244 |
| 374 | BOL | Villa Tunari - Bolívia | -16.97043 | -65.21001 |
| 375 | BOL | San Pedro - Bolívia | -14.42390 | -64.86053 |
| 376 | BOL | Limoncito - Bolívia | -18.24663 | -59.91882 |
| 377 | BOL | Yacuces - Bolívia | -18.98723 | -58.24453 |
| 378 | BOL | Los Volcanes - Bolívia | -18.10037 | -63.59343 |
| 379 | VEN | Tucacas - Venezuela | 10.86081 | -68.33140 |
| 380 | VEN | Churaguara - Venezuela | 10.67215 | -69.24857 |
| 381 | VEN | San Filipi - Venezuela | 10.59770 | -68.64485 |
| 382 | VEN | Yurubi - Venezuela | 10.36432 | -68.74982 |
| 383 | VEN | San Sabastian - Venezuela | 10.40245 | -68.00039 |
| 384 | JAM | Windsor Forest - Jamaica | 18.11114 | -76.34124 |
| 385 | JAM | Ginger House - Jamaica | 18.05814 | -76.41414 |
| 387 | JAM | School of Agric. Port Antonio - Jamaica | 18.19923 | -76.48380 |
| 388 | JAM | St. Ann's - Jamaica | 18.38411 | -77.27663 |
| 389 | HON | Lancetilla Botanical - Honduras | 15.74626 | -87.45343 |
| 390 | HON | San Juan Bosque - Honduras | 15.64030 | -87.17666 |
| 391 | HON | Coyolito - Honduras | 13.31492 | -87.62271 |
| 392 | HON | Santa Barbara - Honduras | 12.46915 | -87.58688 |
| 393 | BLZ | Punta Gorda - Belize | 16.09112 | -88.80841 |
| 394 | BLZ | Jaguar Reef Lodge - Belize | 16.83997 | -88.27174 |
| 395 | BLZ | Mayflower Natl Park - Belize | 16.93243 | -88.38291 |
| 396 | NIC | Ometepe - Nicaragua | 11.52188 | -85.71052 |
| 397 | NIC | Road to Cardenas - Nicaragua | 11.23548 | -85.59799 |
| 398 | NIC | Lake Masaya - Nicaragua | 11.97336 | -86.12502 |
| 399 | GTM | San Carlos - Guatemala | 15.78895 | -88.84182 |
| 400 | GTM | Livingston beach - Guatemala | 15.82811 | -88.74756 |
| 401 | GTM | Cerro San Gil Protected Area - Guatemala | 15.68554 | -88.64486 |
| 402 | MEX | Mexico | 19.23112 | -90.84244 |
| 403 | MEX | Coba – Mexico | 20.48872 | -87.73501 |
| 405 | TT | PAX upper trail - Trinidad e Tobago | 10.66400 | -61.40400 |
| 406 | TT | Grand Riviere - Trinidad e Tobago | 10.83000 | -61.04400 |
| 407 | BRW | Dania – Broward | 26.06902 | -80.16941 |
| 408 | BRW | Dania Beach - Broward | 26.06403 | -80.17375 |
| 409 | BHM | Treasure Cay resort - Bahamas | 26.67635 | -77.28432 |
| 410 | BHM | Treasure Cay resort - Bahamas | 26.67643 | -77.28560 |
| 411 | STL | Coconut Bay Resort - St. Lucia | 13.73000 | -60.94000 |
| 412 | SKN | Church Grounds - St Kitts-Nevis | 17.13422 | -62.60183 |
| 413 | DOM | Crompton Point - Dominica | 15.58333 | -61.31667 |
| 415 | UST | Highway 902 - U.S. Terr. | 18.10167 | -65.91272 |
| 416 | CRICA | La Selva Biological Station - Costa Rica | 10.42540 | -84.00220 |
| 417 | CAY | Fountain Court - Ilhas Cayman | 19.28423 | -81.38697 |
| 418 | RDOM | Enriquillo - Rep. Dominicana | 18.00078 | -71.35010 |
| 419 | GRAN | Jessamine Vale - Granada | 12.09006 | -61.73523 |
| 420 | GUA | Highway D23 - Guadalupe | 16.18393 | -61.77020 |
| 437-438 | MG | Ipaba - MG | -19.4623 | -42.43 |
| 439-440 | MG | Caratinga - MG | -19.4562 | -42.3004 |
| 441-442 | MG | Ipanema - MG | -19.7245 | -41.8028 |
| 443-444; 455 | MG | Ipanema - MG | -19.6985 | -41.8241 |
| 445 | MG | Penha do Capim - MG | -19.6284 | -41.2483 |
| 446 | ES | Itapina - ES | -19.5118 | -40.8472 |
| 447 | ES | Colatina - ES | -19.5099 | -40.5591 |
| 448 | ES | Sooretama - ES | -18.9897 | -40.038 |
| 449 | ES | Sooretama - ES | -18.9967 | -40.1267 |
| 450 | ES | Aracruz - ES | -19.821 | -40.1537 |
| 451 | ES | Fundão - ES | -20.0372 | -40.1769 |
| 452 | ES | São José do Calçado - ES | -21.0575 | -41.657 |
| 453 | ES | São José do Calçado - ES | -21.5 | -42.083333 |
| 454 | MG | São Geraldo - MG | -21.8315 | -42.6069 |
| 456 | GO | Goiânia - GO | -16.5843 | -49.1614 |
| 469 | PR | Céu Azul - PR | -25.175126 | -53.913901 |
| 470 | PR | Ivailândia - PR | -23.710449 | -52.160627 |
| 471 | PR | Floresta - PR | -23.598891 | -52.07929 |
| 472-473 | PR | Maringá - PR | -23.497496 | -52.014919 |
| 474 | PR | Maringá - PR | -23.279694 | -51.888869 |
| 475 | PR | Santa Fé - PR | -23.056231 | -51.82309 |
| 476 | PR | Nossa Senhora das Graças - PR | -22.882321 | -51.790357 |
| 477 | SP | Itororó - SP | -22.580632 | -51.707383 |
| 478 | SP | Tarabaí - SP | -22.366O206 | -51.583056 |
| 479 | SP | Pirapozinho - SP | -22.271347 | -51.484303 |
| 480 | SP | Presidente Prudente - SP | -22.173129 | -51.344981 |
| 481 | SP | Martinópolis - SP | -22.081654 | -51.12027 |
| 482 | SP | Osvaldo Cruz - SP | -21.874343 | -50.8583 |
| 483 | SP | Olímpia - SP | -20.72184 | -48.869361 |
| 487-495; 502-509 | BA | Entre Rios - BA | -11.9419444 | -38.08444 |
| 496-501 | BA | Alagoinhas - BA | -12.13555 | -38.419166 |
| 511 | ES | Colatina - ES | -19.5099 | -40.5591 |
| 512 | ES | Sooretama - ES | -18.9897 | -40.038 |
| 513 | SP | Ribeirão Preto - SP | -21.225277 | -47.8272222 |
| 515 | SP | Ribeirão Preto - SP | -21.178055 | -47.78 |
| 583 | GO | Ivolândia - GO | -16.6101 | -50.9525 |
| 584 | MG | Paracatu - MG | -17.2108 | -46.8956 |
| 585 | MT | Araguainha - MT | -16.7994 | -52.9446 |
| 586 | GO | Firminópolis - GO | -16.5697 | -50.3082 |
| 587 | MG | Paracatu - MG | -16.8409 | -53.027 |
| 588 | MS | Paraíso das Águas - MS | -19.0462 | -53.0096 |
| 589 | GO | Ivolândia - GO | -16.6068 | -50.9086 |
| 590 | MT | Pedra Preta - MT | -16.6068 | -54.4709 |
| 591 | MG | Paracatu - MG | -17.1896 | -46.9226 |
| 592 | GO | Piranhas - GO | -16.4256 | -51.8316 |
| 593 | MS | Coxim - MS | -18.495 | -54.6354 |
| 594 | MS | Campo Grande - MS | -20.4877 | -54.8131 |
| 595 | MS | Antônio João - MS | -22.2159 | -55.9236 |
| 596 | MS | Perdido - MS | -21.7419 | -57.1254 |
| 597 | MS | Camapuã - MS | -19.5211 | -54.0406 |
| 598 | MS | Bandeirantes - MS | -19.8639 | -54.3688 |
| 599 | MS | Costa Rica - MS | -18.5641 | -53.121 |
| 600 | MT | Pedra Preta - MT | -16.549 | -54.5748 |
| 601 | MS | Dourados - MS | -22.2334 | -54.5815 |
| 602 | MS | Ivinheima - MS | -22.3337 | -53.7695 |
| 603 | MS | Anastácio - MS | -20.7137 | -55.9087 |
| 604 | GO | Diorama - GO | -16.3947 | -51.2571 |
| 605 | PB | João Pessoa - PB | -7.13445 | -34.84602 |
| 606 | PB | Mamanguape - PB | -6.7416 | -35.1405 |
| **Total: 230 individuals** | **-** | **180 localities** | - | - |

* *GenBank* access number.
