# Supplementary material for: Phylogeography of Nasutitermes corniger (Isoptera: Termitidae) in the Neotropical Region
Source: BMC Evol Biol. 2017 Nov 23;17:230. doi: 10.1186/s12862-017-1079-8 (PMC5701342; doi:10.1186/s12862-017-1079-8)
Supplement: Supplementary file 2 — Details of observed Nasutitermes corniger haplotypes. Relationship among haplotypes, their frequency, sharing with N. corniger specimens, and allocation to haplogroup. Letters preceding voucher numbers (final column) correspond to the respective location code. (DOCX 19 kb) [file 12862_2017_1079_MOESM2_ESM.docx]

Details about haplotypes found in this work.

| **Haplogroup** | **Haplotype** | **Frequency** | **Specimens containing the haplotype** |
| --- | --- | --- | --- |
| **1** | I | 21 | RDOM194, DOM201, CE299, GO300, MA301, MA303, MA304, TO309, PMA361, PMA362, PMA363, HON389, HON390, HON391, HON392, NIC396, NIC397, NIC398, DOM413, GO456, GO589 |
|  | II | 1 | PRU371 |
|  | III | 2 | PRICO195, UST415 |
|  | IV | 1 | SUR200 |
|  | V | 1 | TT405 |
|  | VI | 1 | MS593 |
|  | VII | 1 | GRAN419 |
|  | VIII | 1 | TT406 |
|  | IX | 4 | JAM384, JAM385, JAM387, JAM388 |
|  | X | 4 | VEN379, VEN380, VEN381, VEN383 |
|  | XI | 1 | VEN382 |
|  | XII | 2 | PA306, BA506 |
|  | XIII | 3 | WIND203, BRW407, BRW408 |
|  | XIV | 4 | GUA198, SKN199, SKN412, GUA420 |
| **2** | XV | 11 | MEX197, EQUA202, MA302, BLZ393, BLZ394, BLZ395, GTM399, GTM400, GTM401, MEX402, MEX403 |
|  | XVI | 1 | STL411 |
|  | XVII | 4 | PMA360, BHM409, BHM410, CRICA416 |
|  | XVIII | 3 | PMA358, PMA359, CAY417 |
|  | XIX | 1 | PMA364 |
|  | XX | 1 | RDOM418 |
| **3** | XXI | 19 | PR267, PR268, PR269, MT270, MT271, MT272, MT273, MT274, MT275, MT276, MT277, MT278, MT279, PRU370, PRU372, BOL373, BOL374, BOL375, BOL376 |
|  | XXII | 9 | RO165, RO170, RO171, RO173, RO174, RO175, RO176, RO177, RO178 |
|  | XXIII | 1 | RO172 |
|  | XXIV | 2 | RO166, RO167 |
|  | XXV | 1 | RO168 |
|  | XXVI | 1 | BOL378 |
|  | XXVII | 4 | PGUAI352, PGUAI353, PGUAI354, PGUAI357 |
|  | XXVIII | 5 | MS180, MS182, BOL377, MS596, MS603 |
|  | XXIX | 4 | MS179, MS181, MS184, MS185 |
|  | XXX | 4 | PRU365, PRU366, PRU367, PRU368 |
| **4** | XXXI | 8 | RO156, RO158, RO159, RO162, RO163, RO164, TO310, TO311 |
|  | XXXII | 1 | PRU369 |
|  | XXXIII | 1 | RO161 |
|  | XXXIV | 25 | PGUAI355, PGUAI356, PR469, PR470, PR471, PR472, PR473, PR474, PR475, PR476, SP477, SP478, SP479, SP480, SP481, MT585, MS588, MS594, MS595, MS597, MS598, MS599, MT600, MS601, MS602 |
|  | XXXV | 1 | MS183 |
|  | XXXVI | 1 | MT590 |
| **5** | XXXVII | 22 | JAB88, JAB89, JAB90, JAB91, JAB92, JAB93, JAB94, JAB95, PB281, PB282, MG283, PB298, MG347, MG348, SE350, TAN351, SP482, SP483, SP513, SP515, MG587, PB606 |
|  | XXXVIII | 5 | GO583, MG584, GO586, MG591, GO604 |
|  | XXXIX | 1 | PA307 |
|  | XL | 1 | PB280 |
|  | XLI | 1 | AL349 |
| **6** | XLII | 36 | MG437, MG438, MG439, MG440, MG441, MG442, MG443, MG444, MG445, ES446, ES447, ES448, ES449, ES450, ES451, ES452, RJ453, MG454, MG455, BA487, BA490, BA491, BA492, BA493, BA494, BA495, BA498, BA502, BA503, BA504, BA505, BA507, ES511, ES512, GO592, PB605 |
|  | XLIII | 6 | BA496, BA499, BA500, BA501, BA508, BA509 |
|  | XLIV | 2 | BA488, BA489 |
|  | XLV | 1 | PB297 |
