# Supplementary material for: Phylogeography of Nasutitermes corniger (Isoptera: Termitidae) in the Neotropical Region
Source: BMC Evol Biol. 2017 Nov 23;17:230. doi: 10.1186/s12862-017-1079-8 (PMC5701342; doi:10.1186/s12862-017-1079-8)
Supplement: Supplementary file 3 — Haplotypic network evidencing the nested clades used in the NCPA. Each line of the network represents a single mutation step. Haplotypes are represented by Roman numerals; small circles indicate hypothetical haplotypes that are necessary intermediates between the haplotypes observed. Dotted lines indicate level 1 clades; dashed lines indicate level 2 clades; full clear lines indicate level 3 clades; full dark lines indicate level 4 clade. (PDF 218 kb) [file 12862_2017_1079_MOESM3_ESM.pdf]

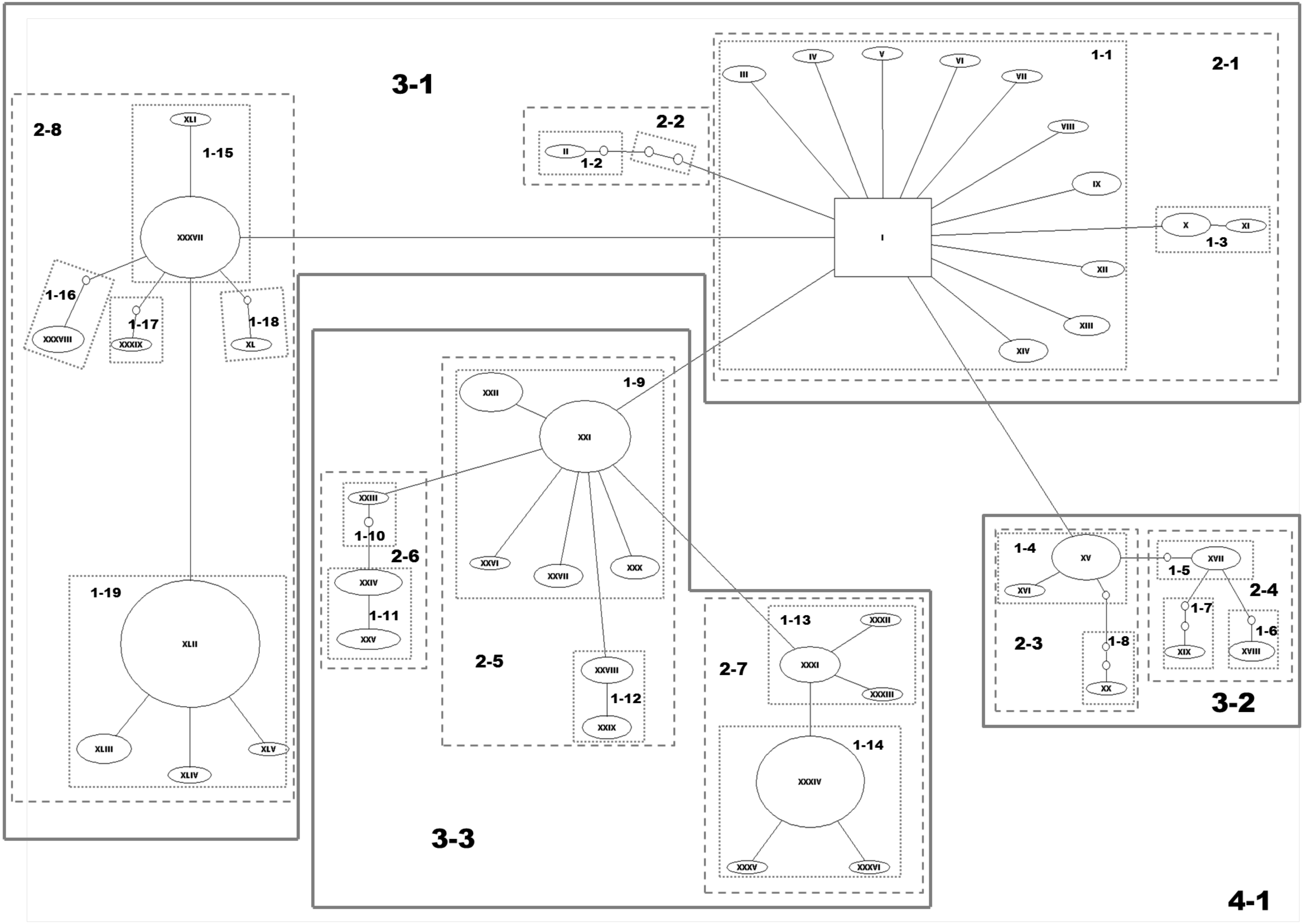

3-1
2-8
XLI
1-15
XXXVII
1-16
XXXVIII
1-17
XXXIX
1-18
XL
1-19
XLII
XLIII
XLIV
XLV
2-2
II
1-2
1-1
III
IV
V
VI
VII
VIII
IX
I
X
XI
1-3
XII
XIII
XIV
2-1
3-3
1-9
XXII
XXI
XXIII
1-10
2-6
XXIV
1-11
XXV
XXVI
XXVII
XXX
2-5
XXVIII
1-12
XXIX
2-7
1-13
XXXII
XXXI
XXXIII
1-14
XXXIV
XXXV
XXXVI
1-4
XV
XVI
1-5
XVII
2-4
1-7
1-6
2-3
1-8
XIX
XVIII
XX
3-2
4-1
